# HEARING LOSS AND SOCIAL ISOLATION: THE ROLE OF NEIGHBORHOOD SOCIAL COHESION AND DISORDER

**DOI:** 10.1093/geroni/igz038.3462

**Published:** 2019-11-08

**Authors:** Sol Baik, Amanda Lehning

**Affiliations:** 1 University of Maryland, Baltimore, Baltimore, Maryland, United States; 2 University of Maryland, Baltimore, Maryland, United States

## Abstract

Social isolation is a critical public health issue that socially isolated individuals are at increased risk for mortality and deteriorated health. Those who acquire hearing loss in later life experience difficulties with communication, potentially leading to social isolation. However, less is known about the social consequences of age-related hearing loss, and few studies have assessed the influence of environmental factors on hearing loss and social isolation. The aims of this study are to examine: (1) the association between hearing loss and social isolation of older adults over time, and (2) the moderating effects of perceived neighborhood social cohesion and disorder on this relationship. We analyzed 2,080 community-dwelling Medicare beneficiaries aged 65 or above from Round 1 to 3 of National Health and Aging Trends Study. We conducted random coefficient models, entering hearing loss as a random coefficient. Older adults with hearing loss were less socially isolated than those without hearing loss. However, the effect of hearing loss on social isolation varied depending on perceived neighborhood social cohesion. Older adults with hearing loss who reported high neighborhood cohesion had significantly lower social isolation compared to those without hearing loss, while those with hearing loss who perceived low social cohesion had significantly higher social isolation. Our findings suggest neighborhood social cohesion can serve as a potential protective factor for older adults with hearing loss. This poster will propose neighborhood-level interventions that could supplement other services for those with hearing loss, such as assistive devices that are rarely covered by health insurance.

